# Root Maturity as a Determinant of Post-traumatic Endodontic Outcomes: A Comparative Case Report

**DOI:** 10.7759/cureus.98490

**Published:** 2025-12-04

**Authors:** Nevia Pradeep, Jemi Wilson, Parvathy Kumaran, Balagopal Varma R, Suresh Kumar J, Arun Mamachan Xavier, Malini Venugopal, Nishna Thankappan, Cherupally K Krishnan Nair, Sheena P Kochumon

**Affiliations:** 1 Pediatric Dentistry, Amrita Vishwa Vidyapeetham, Kochi, IND; 2 Health Sciences, Amrita Vishwa Vidyapeetham, Kochi, IND; 3 Pediatric and Child Health/Genomics/Health Sciences and Research, Amrita Vishwa Vidyapeetham, Kochi, IND

**Keywords:** closed apex, dental trauma, external inflammatory resorption, open apex, pediatric dentistry, revascularization, root resorption, tooth replantation

## Abstract

Tooth avulsion and intrusion are severe traumatic dental injuries that often lead to tooth loss and root resorption. The main determinants of prognosis include root maturity, extra-alveolar time, and the timing and type of treatment. This study presents a comparative analysis of the healing responses in two pediatric cases following delayed and complicated replantation, with a focus on how the maturity of the tooth root influenced the outcomes.

The case report details two pediatric cases with significantly different healing trajectories after replantation. Case 1 involves an eight-year-old boy with an avulsed immature permanent left central incisor (21) that was replanted after approximately six hours of extra-oral time. A revascularization procedure was performed two weeks post-replantation. While limited external resorption was noted at three months, it spontaneously arrested by the six-month follow-up. Case 2 presents a 10-year-old boy with severe intrusion of mature maxillary central incisors (11, 21), which were surgically repositioned and replanted. Root canal treatment was initiated at the second week, but radiographic evidence of progressive resorption was observed on both teeth by the end of six weeks.

The immature tooth (Case 1) demonstrated a favorable healing response, characterized by revascularization and the spontaneous arrest of resorption. In contrast, the mature teeth (Case 2) experienced ongoing, progressive resorption despite conventional endodontic intervention.

Root maturity significantly influences the regenerative potential and resorption outcome in replanted teeth. Immature teeth with open apices possess a greater capacity for healing and may benefit from regenerative endodontic approaches, even in instances of delayed replantation. Conversely, mature teeth with closed apices have a more guarded prognosis, with a higher propensity for progressive resorption following severe traumatic injuries.

## Introduction

Traumatic dental injuries (TDIs) are a common occurrence in children and adolescents, with tooth avulsion and intrusion representing two of the most severe forms [1]. TDIs affect approximately 20%-30% of children, making them a major public health concern and a frequent cause of long-term dentoalveolar complications. It can significantly influence long-term occlusal development, orthodontic treatment needs, and dental maturation, as highlighted by Alam et al., who demonstrated that early dentoalveolar trauma alters future orthodontic parameters and craniofacial growth trajectories [2]. The management of such injuries is complex, and the prognosis is often uncertain, with outcomes heavily influenced by a variety of factors. The International Association of Dental Traumatology (IADT) has established comprehensive guidelines for the management of avulsed permanent teeth, emphasizing the critical importance of prompt and appropriate emergency care to maximize the chances of a favorable outcome [3].

The primary goal of replantation is to preserve the vitality of the periodontal ligament (PDL) cells, which is crucial for preventing root resorption and ensuring long-term tooth survival [4]. The extra-alveolar time (EAT), particularly the duration of dry time, and the choice of storage medium are critical determinants of PDL cell viability [5]. While immediate replantation is ideal, this is not always feasible. In cases of delayed replantation, where the EAT exceeds 60 minutes, the PDL is generally considered non-viable, and the prognosis becomes significantly poorer [6].

Following replantation, teeth are at high risk for several complications, like root resorption. Andreasen classified the outcomes of PDL healing into four types: healing with a normal PDL, surface resorption, inflammatory resorption, and replacement resorption (ankylosis) [4]. A meta-analysis by Souza et al. found a high incidence of resorption, with replacement resorption (ankylosis) being the most common type (51.0%), followed by inflammatory resorption (23.2%) [1]. The pathophysiological mechanisms underlying these processes are complex, initiated by damage to the protective cementum and predentine layers, which exposes the underlying dentin to cellular activity [7].

Another critical factor influencing the prognosis is the stage of root development at the time of injury. More favorable healing outcomes are there for immature teeth with open apices, having a recognized potential for revascularization and continued root development [8]. This contrasts with mature teeth with closed apices, which have a more limited healing capacity and a higher incidence of complications such as pulp necrosis and resorption, particularly following severe intrusion injuries [9]. Adjunctive methods, such as low-level laser therapy, have been explored to mitigate resorption risk after intrusion, although their efficacy remains under investigation [10]. Current evidence supports moderate-quality outcomes for regenerative endodontic procedures in traumatized immature teeth, although long-term data remain limited.

This comparative case report aims to illustrate the profound influence of root maturity on the healing outcomes of two distinct cases of severe dental trauma. By presenting a case of delayed replantation of an immature tooth alongside a case of severe intrusion of mature teeth, we highlight the different biological responses and discuss the implications for clinical management and prognosis.

## Case presentation

Case report 1

Patient Information and Clinical Findings

An eight-year-old boy presented to our department with an avulsed permanent left central incisor (21) following a fall from a bicycle. The tooth had been kept in a container of milk during the extra-oral period, which was estimated to be approximately 5-6 hours. The patient was systemically healthy with no reported allergies or relevant medical history. Intraoral examination revealed a clean, empty socket for tooth 21 with no signs of alveolar fracture.

Therapeutic Intervention

**Figure 1:**
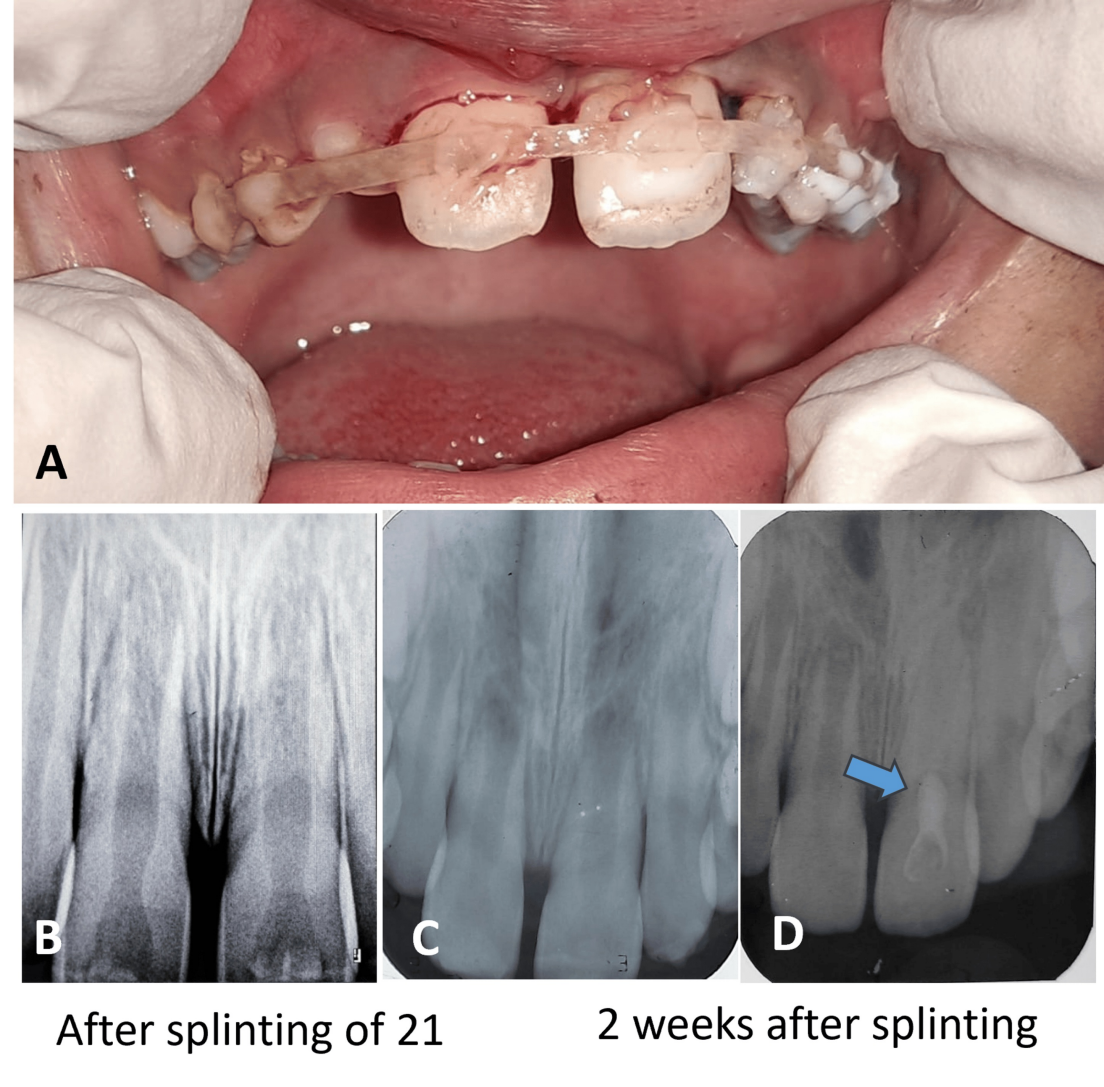
Clinical and radiographic images of splinted tooth 21, with revascularization done at 2 weeks (A, B) Immediately after splinting; (C, D) revascularization done at the 2-week follow-up

The avulsed tooth, which had an immature root with an open apex, was gently rinsed with saline and replanted into the socket under local anesthesia. A flexible splint, using a fiber-reinforced composite, was placed to stabilize the tooth from the right canine (13) to the left canine (23) (Figures 1A, 1B). Due to the prolonged extra-oral time and the high risk of pulp necrosis, a revascularization procedure was planned for later.

Two weeks after replantation, the revascularization procedure was initiated. The tooth was isolated, and after accessing the pulp chamber, necrotic tissue was removed. The canal was gently irrigated with 1% sodium hypochlorite and 17% ethylenediaminetetraacetic acid (EDTA) (Figure 1C). After inducing bleeding, placement of a mineral trioxide aggregate (MTA) plug was done to create a hermetic seal and encourage tissue regeneration and root development (Figure 1D).

Follow-Up and Outcomes

**Figure 2:**
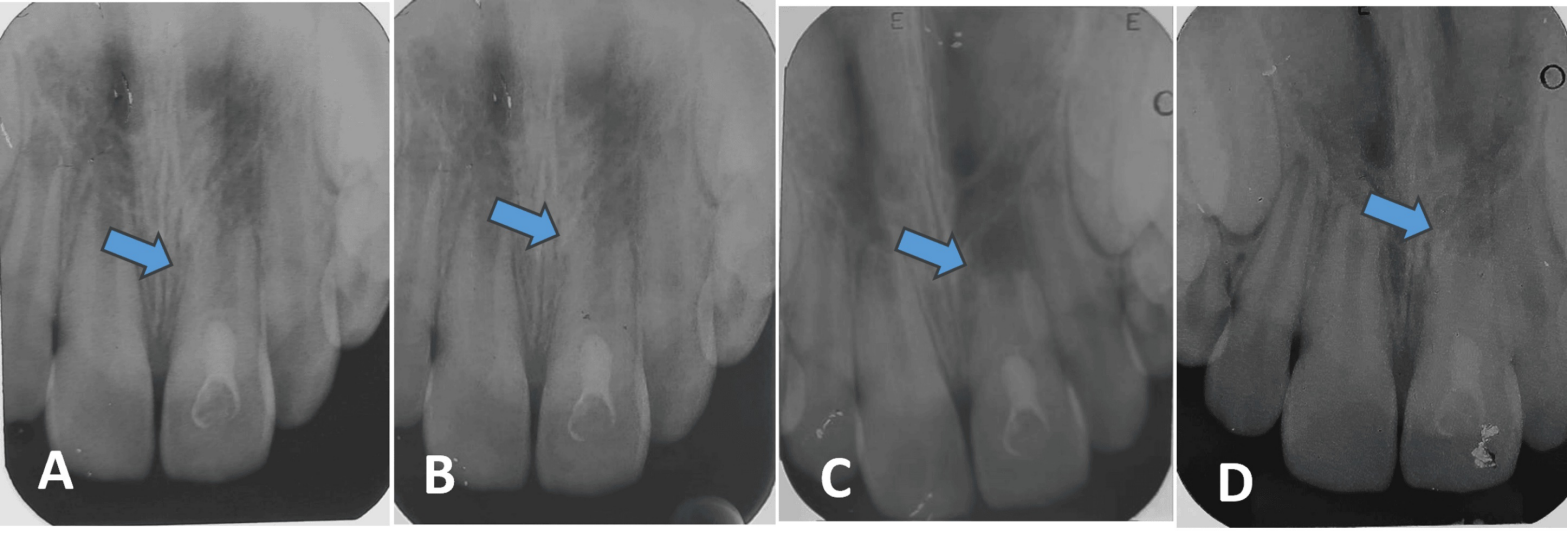
Follow-up timeline radiographs (A) At 3 months - initiation of limited external inflammatory resorption on the mesial aspect of the root - inflammatory resorption (Andreasen PDL Healing Classification); (B) at 6 months - the resorptive process had spontaneously arrested, indicating progression to functional healing; (C) at 9 months; (D) at 12 months - the root length appeared stable PDL: periodontal ligament

At the three-month follow-up, radiographic evaluation revealed the initiation of limited external inflammatory resorption on the mesial aspect of the root (Figure 2A), classified as inflammatory resorption (Andreasen PDL Healing Classification). However, by the six-month review, the resorptive process had spontaneously arrested, and the root length appeared stable (Figures 2B, 2C), indicating progression to functional healing. The tooth remained asymptomatic and functional, with evidence of apical closure (Figure 2D). This outcome was considered favorable, especially given the significant delay in replantation.

Case report 2

Patient Information and Clinical Findings

**Figure 3:**
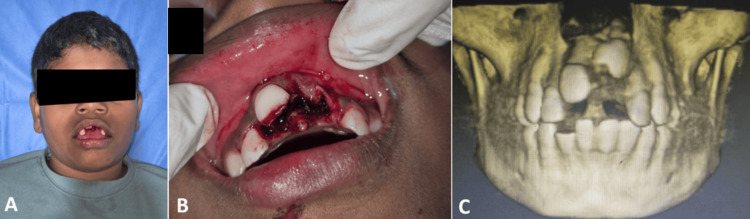
(A, B) Clinical presentation at the time of trauma; (C) 3D view (CBCT scan) CBCT: cone-beam computed tomography

A 10-year-old boy was referred following a road traffic accident, resulting in severe intrusion of the maxillary central incisors (11 and 21) (Figures 3A, 3B). The patient had a known medical history of rheumatoid arthritis. Clinical examination revealed that tooth 21 was intruded by more than 10 mm, while tooth 11 was intruded by approximately 3-7 mm (Figure 3C). There was also an associated Ellis Class III fracture of the mandibular right lateral incisor (42) and multiple soft tissue ulcerations. Cone-beam computed tomography (CBCT) has demonstrated superior diagnostic accuracy for evaluating intrusion depth, PDL breakdown, and early resorptive defects compared with 2D radiographs, supporting its use in severe trauma cases (Figure 3C) [11].

Therapeutic Intervention

**Figure 4:**
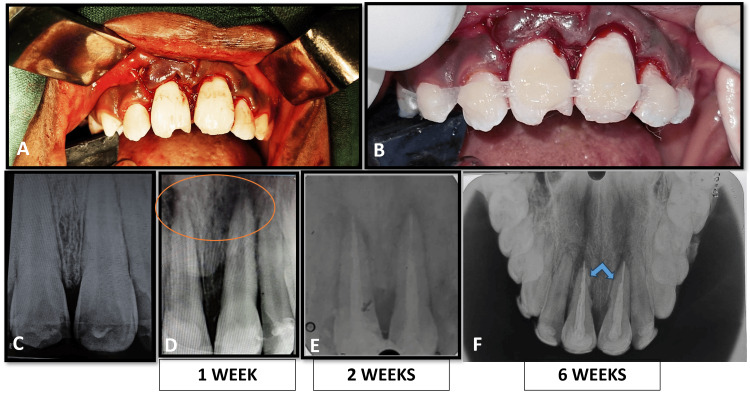
Follow-up timeline of progressive external inflammatory resorption (A, B) Clinical images immediately after repositioning and splinting. (C) Radiographic image of repositioned teeth 11 & 21. (D) A large, diffuse radiolucency noted at the apical region of both intruded incisors at the 1-week follow-up, indicative of pulpal and periapical inflammation. (E) Conventional root canal treatment performed at the 2nd week. (F) At 6 weeks post-treatment, clear evidence of progressive external inflammatory resorption on the roots of both central incisors was revealed - inflammatory resorption (Andreasen PDL Healing Classification) PDL: periodontal ligament

Given the severity of the intrusion and the patient's age, surgical repositioning of the intruded teeth was performed under general anesthesia. The teeth were carefully extruded and repositioned to their correct alignment (Figures 4A, 4C). A fiber-reinforced composite splint was applied, extending from the right canine (13) to the left canine (23), to ensure stabilization (Figure 4B).

At the one-week follow-up, a large, diffuse radiolucency was noted at the apical region of both intruded incisors, indicative of pulpal and periapical inflammation (Figure 4D). Due to the mature status of the roots (closed apices) and the high likelihood of pulp necrosis following such a severe injury, conventional root canal treatment was initiated at the second week. A single-visit root canal therapy was performed on both teeth 11 and 21 (Figure 4E), and the fiber splint was removed.

Follow-Up and Outcomes

Despite the endodontic intervention, radiographic examination at six weeks post-treatment revealed clear evidence of progressive external inflammatory resorption on the roots of both central incisors (Figure 4F), classified as inflammatory resorption according to the Andreasen PDL Healing Classification. The teeth, although stable, showed a guarded prognosis due to the ongoing resorptive process. The patient was placed on a periodic recall schedule for close monitoring of the resorption and to manage any further complications.

Comparison of cases

The two cases presented in Table 1 offer a stark contrast in healing outcomes, which can be attributed to several key differentiating factors. A direct comparison highlights the critical role of root maturity in the prognosis of severely traumatized permanent teeth.

**Table 1:** Comparison of Cases 1 & 2 (favorable vs. unfavorable outcomes) Functional healing vs. progressive inflammatory resorption (Andreasen PDL Healing Classification) EAT: extra-alveolar time; PDL: periodontal ligament

Feature	Case 1 (favorable outcome)	Case 2 (unfavorable outcome)
Patient age	8 years	10 years
Tooth/teeth	21 (maxillary left central incisor)	11, 21 (maxillary central incisors)
Root maturity	Immature (open apex)	Mature (closed apex)
Type of injury	Avulsion	Severe intrusion (>7 mm)
Initial management	Delayed replantation (~6 hours EAT)	Surgical repositioning
Endodontic treatment	Regenerative (revascularization) at 2 weeks	Conventional root canal treatment at 2 weeks
Resorption outcome	Limited external resorption, spontaneously arrested, progression to functional healing	Progressive inflammatory resorption
Prognosis	Favorable, with tooth retention and stable root length	Guarded, with ongoing resorption and risk of tooth loss

## Discussion

The divergent outcomes of these two cases elevate the profound impact of root maturity on the healing process following severe dental trauma. According to Andreasen, PDL healing after replantation can be classified into four categories: healing with a normal PDL, healing with surface resorption, healing with inflammatory resorption, and healing with replacement resorption (ankylosis) [4]. The favorable result in Case 1, despite a significant delay in replantation, can be largely attributed to the biological potential of the immature tooth. The open apex allowed for the successful application of a revascularization procedure, a regenerative endodontic technique that aims to re-establish a vital pulp-like tissue within the root canal space [8]. The spontaneous arrest of resorption observed in this case is a testament to the remarkable healing capacity of the developing periodontium and pulp in young patients. This finding is consistent with literature suggesting that immature teeth have a greater chance of pulpal and periodontal healing, even in challenging clinical scenarios [4,9].

The success of revascularization in Case 1, despite an extra-oral time of nearly six hours, is particularly noteworthy. While the IADT guidelines recommend immediate replantation and consider an EAT of over 60 minutes to be a significant negative prognostic factor, this case suggests that regenerative approaches can, in some instances, overcome the limitations of delayed replantation [3,5]. The subsequent revascularization procedure may have provided the necessary biological stimulus to promote repair and halt the resorptive process that had initiated.

In contrast, Case 2 illustrates the challenging nature of managing severe intrusive luxation in mature permanent teeth. Intrusion is considered one of the most severe forms of dental trauma, often leading to extensive damage to the PDL, cementum, and neurovascular supply [9,12]. The mature status of the teeth, with their closed apices, precluded any possibility of revascularization. The only viable option was conventional root canal treatment to address the inevitable pulp necrosis [13].

However, as demonstrated in this case, endodontic treatment alone is often insufficient to prevent or arrest trauma-induced external root resorption. The surgical repositioning, while necessary to restore the teeth to their correct position, likely caused additional trauma to the already compromised PDL. Interventions that can influence root resorption risk, such as alveolar corticotomy, have been studied in other contexts like orthodontic-induced root resorption, but their applicability in trauma cases is not yet established [14]. This combination of factors created an environment ripe for progressive inflammatory resorption, which was observed radiographically just six weeks after the incident. This outcome aligns with the findings of a meta-analysis by Souza et al., which reported an incidence of inflammatory resorption (23.2%) in replanted teeth [1]. The study by Andreasen et al. on 400 avulsed teeth also highlighted that mature teeth have a significantly lower chance of periodontal healing compared to immature teeth [4].

Even with a poor prognosis in fractured teeth with mature root apices, endodontic treatment remains compulsory to prevent further complications and maintain the tooth structure for as long as possible. It is imperative that patients and caregivers are thoroughly instructed about the future prognosis, including potential complications such as tooth discoloration, progressive resorption, and eventual tooth loss. Biomechanical and inflammatory responses associated with intrusive or orthodontic forces have a known capacity to accelerate resorption; Alam et al. reported that alterations in alveolar bone turnover can significantly influence susceptibility to root resorption [2]. Despite these challenges, the benefits of retaining the natural tooth - such as maintaining alveolar bone, preserving space, and supporting adjacent teeth - justify the intervention, even when the long-term prognosis is guarded [15].

## Conclusions

The comparative case report of these two cases provides compelling clinical evidence for the critical role of root maturity in determining the prognosis of severely traumatized and replanted permanent teeth. The immature tooth with an open apex demonstrated a remarkable capacity for healing and spontaneous arrest of resorption, even with a significant delay in replantation, highlighting the potential of regenerative endodontic procedures in such scenarios. Conversely, the mature teeth with closed apices had a much poorer outcome, with progressive and uncontrolled resorption despite timely and conventional endodontic treatment. Despite the guarded prognosis associated with mature root apices, it is strongly advisable to attempt tooth retention through endodontic intervention. Patients and caregivers must be clearly informed about the poor prognosis and potential complications, including tooth discoloration and progressive resorption.

This report underscores the importance of considering the biological status of the root as a primary factor in treatment planning and prognostic assessment for TDIs in the pediatric population. Further research is warranted to explore the full potential of regenerative therapies in expanding the window for successful outcomes in cases of delayed replantation of immature teeth.
